# The Need for Telehealth Education in the Medical School Curriculum

**DOI:** 10.7759/cureus.90598

**Published:** 2025-08-20

**Authors:** Madilyn Carney, Mary E Thornton, Kavya Avancha, James R Nolin, Julia Alexander

**Affiliations:** 1 Medical School, Alabama College of Osteopathic Medicine, Dothan, USA; 2 Radiology, Alabama College of Osteopathic Medicine, Dothan, USA

**Keywords:** medical school curriculum, medical student survey, telehealth challenges, telehealth education, telemedicine experience

## Abstract

The need for telehealth increased significantly during the COVID-19 pandemic and has continued to be a popular method for patients to consult with their physicians. As telemedicine has expanded and become an integral part of a physician's daily practice, a question arises: how does one translate an in-person visit to a virtual one? Therefore, we proposed a research study to assess the need to incorporate more telehealth education into the medical school curriculum. We began by surveying third- and fourth-year medical students at Alabama College of Osteopathic Medicine (ACOM) to see how often they conducted or were exposed to telehealth visits during their clinical rotations. In addition, we also assessed their confidence in performing a telehealth visit, along with which part of the clinical encounter was most difficult for the students. Out of 368 students surveyed, we received only 43 responses, which is considered low and represents a limitation of our study. However, based on the collected responses, combined with other referenced articles, it is evident that there is a need for a more established addition to the curriculum regarding telehealth education. In this paper, we also proposed a variety of possible solutions to fulfill this need so that future medical students will feel fully prepared when encountering a patient virtually.

## Introduction

Currently, telehealth is one of the fastest-growing sectors in healthcare, with approximately 50% of hospitals in the United States in 2022 engaged in some form of telemedicine [1]. The popularity of physician-to-patient interactions via technology has increased dramatically in the past decade [1]. Telehealth can be categorized into two main formats: asynchronous and synchronous. Asynchronous telehealth involves collecting and transmitting patient data (such as images and medical reports) for later evaluation by a physician without direct communication. In contrast, this study focuses on synchronous telehealth, which entails real-time patient-physician interactions over video calls, phone consultations, or chat platforms [1]. This format of care delivery has made it easier to care for those with mobility and transportation issues.

Telehealth has brought an increased ease of access between patients and physicians that has led to improved adherence to care plans and fewer hospital readmissions [2]. Telehealth has also facilitated improved interprofessional collaboration, enabling physicians to consult with specialists more efficiently [2]. Telehealth allows healthcare professionals to communicate seamlessly, enhancing coordination and efficiency in patient care management [2]. Telehealth is widely utilized in specialties such as primary care, psychiatry, dermatology, and cardiology, where remote consultations can effectively address patient needs [2]. It has also been seen to decrease the cost and time needed to manage many chronic health issues such as diabetes, sleep apnea, and asthma [2].

The COVID-19 pandemic significantly increased the demand for physicians to conduct virtual patient encounters effectively. This need has not diminished, and the benefits of telehealth, such as cost efficiency and the ability to care for people more easily in rural areas, are just a few of the reasons why it remains necessary [3]. Although telehealth offers these benefits, we cannot overlook the limitations of telehealth-based medical care compared to in-person care [4]. Telehealth efficiency can be severely diminished in a multitude of ways, including network outages, misrepresentation of symptoms, a lack of hands-on physical exams, and others. These factors must be acknowledged when learning about telehealth in school and should be considered in conjunction with patient demographics when determining whether telehealth is an adequate mode of healthcare delivery for a specific patient [5].

Given the growing demand for telehealth, we must assess whether medical students receive adequate training in conducting virtual medical evaluations. If not, how can medical school curricula be adapted to bridge this gap? This study aims to assess medical students' exposure to telehealth, their confidence in virtual encounters, and their perspectives on curriculum integration. Based on a recent study, the reported incorporation of telehealth into undergraduate and graduate-level curricula is very little [6]. Therefore, due to the absence of telehealth in the preclinical curricula at the Alabama College of Osteopathic Medicine (ACOM), we hypothesized that most students would not feel prepared to perform virtual encounters and would suggest more integration of telehealth into the curriculum.

## Materials and methods

This study was designed as a survey research project, collecting both qualitative and quantitative data. Data were collected virtually over a 28-day period and analyzed primarily at the ACOM campus in Dothan, AL. The survey consisted of 15 questions created on Google Forms. The questions were written by the authors and determined among themselves to address the pertinent question of whether medical students in their clinical years felt prepared and comfortable using telehealth as a method of healthcare delivery. Furthermore, these questions were written in order to target our sample population of third- and fourth-year osteopathic medical students (OMS) who attend ACOM. In general, the questions created for the survey were not pilot-tested or validated before distribution.

The sampling method used can be described as non-probability and purposive. This particular population was selected based on the availability of contact information for the authors who attended the same program. The eligibility criteria for participating in this survey were limited to being a third- or fourth-year medical student at ACOM in April 2024. Regarding ethical clearance, this project was approved by the ACOM Institutional Review Board in March 2024. The survey was distributed via a faculty ACOM email associated with James Nolin to all 368 OMS III and OMS IV students at ACOM. The email included an attached document stating that, upon completing the survey, participants consent to the use of the information provided in this research study. The survey was determined to have an estimated completion time of under 10 minutes, based on pre-testing among faculty and students. It was disseminated in April 2024, with one reminder email sent approximately three weeks later to improve response rates. The survey remained open for data collection for a total of 28 days.

Survey data were securely stored on password-protected computers accessible only to research team members and the principal investigator (PI). The statistical analysis aimed to summarize and interpret survey responses, using descriptive statistics to evaluate students' exposure to telehealth, confidence levels, and perceptions of curriculum adequacy. The statistical measures employed included the frequencies, percentages, means, and standard deviations of the various survey items. Frequencies and percentages were calculated for demographic variables, such as the year of study (OMS III or OMS IV). The proportion of students exposed to telehealth during their preclinical years was calculated. This was represented as a percentage of the total respondents. The survey assessed the frequency of telehealth encounters during clinical rotations. Data were summarized using frequencies and percentages and represented visually using pie charts. Based on a population size of 368 OMS III and IV students and a 95% confidence interval, a sample size of 188 students was calculated. We utilized a convenience sampling approach, selecting participants based on their availability and willingness to participate during scheduled simulation sessions. The sample consisted of third- and fourth-year medical students, and the data collected reflect the number of responses received from this group. This method was chosen to facilitate timely data collection within the constraints of the academic calendar and simulation program logistics. To evaluate differences in telehealth exposure and perceived preparedness between third- and fourth-year medical students, we conducted statistical comparisons using chi-square and Mann-Whitney U tests. A chi-square test revealed no statistically significant difference between OMS III and OMS IV students in terms of exposure to preceptor-led telehealth encounters during clinical rotations. Additionally, a Mann-Whitney U test showed no significant difference in the number of student-conducted telehealth encounters between OMS III and OMS IV students. Lastly, student-perceived preparedness based on the preclinical curriculum also did not significantly differ between the two groups. These findings suggest a relatively uniform exposure and perceived readiness for telehealth among upper-year medical students, highlighting potential gaps in consistent telehealth education across clinical years.

Before analysis, we reviewed survey data for accuracy and consistency, addressing incomplete responses, duplicates, and inconsistencies. Each response was assigned a unique identifier to maintain anonymity. Students rated their preparedness for conducting telehealth encounters on a five-point Likert scale. The five-point Likert scale increments included strongly disagree, disagree, neutral, agree, and strongly agree. To assess the internal consistency of the Likert scale items measuring students' perceived difficulty with various components of telehealth encounters, we calculated Cronbach's alpha. The scale included items related to history-taking, physical examination, assessment, plan formulation, and establishing patient connections. The analysis yielded a Cronbach's alpha of 0.89, indicating strong internal consistency among the items. This suggests that the scale reliably measures a single underlying construct related to perceived telehealth communication and clinical challenges. The mean and standard deviation of these ratings were calculated to measure central tendency and variability. The difficulty of different components of telehealth encounters (e.g., history-taking, physical exam, assessment/plan, patient connection) was rated on a five-point Likert scale. The mean, standard deviation, and frequency distribution of these ratings were calculated. The survey included whether students believed telehealth education should be incorporated into the curriculum. The responses were summarized using descriptive statistics.

## Results

The survey was sent out to a total of 368 ACOM medical students. Forty-three of the 368 eligible participants completed the survey, which consisted of 21 OMS-IIIs and 22 OMS-IVs.

**Figure 1:**
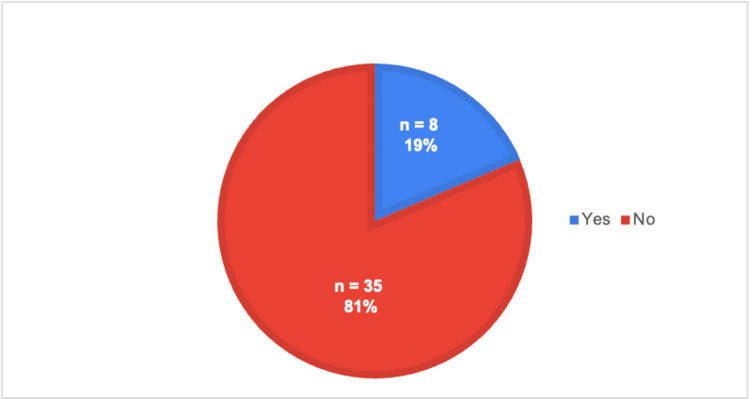
Exposure to telehealth during preclinical years

**Figure 2:**
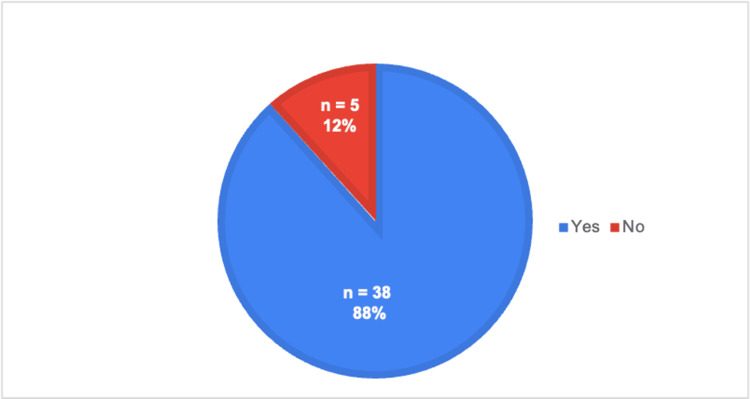
Whether telehealth visits occurred during clinical rotations

**Figure 3:**
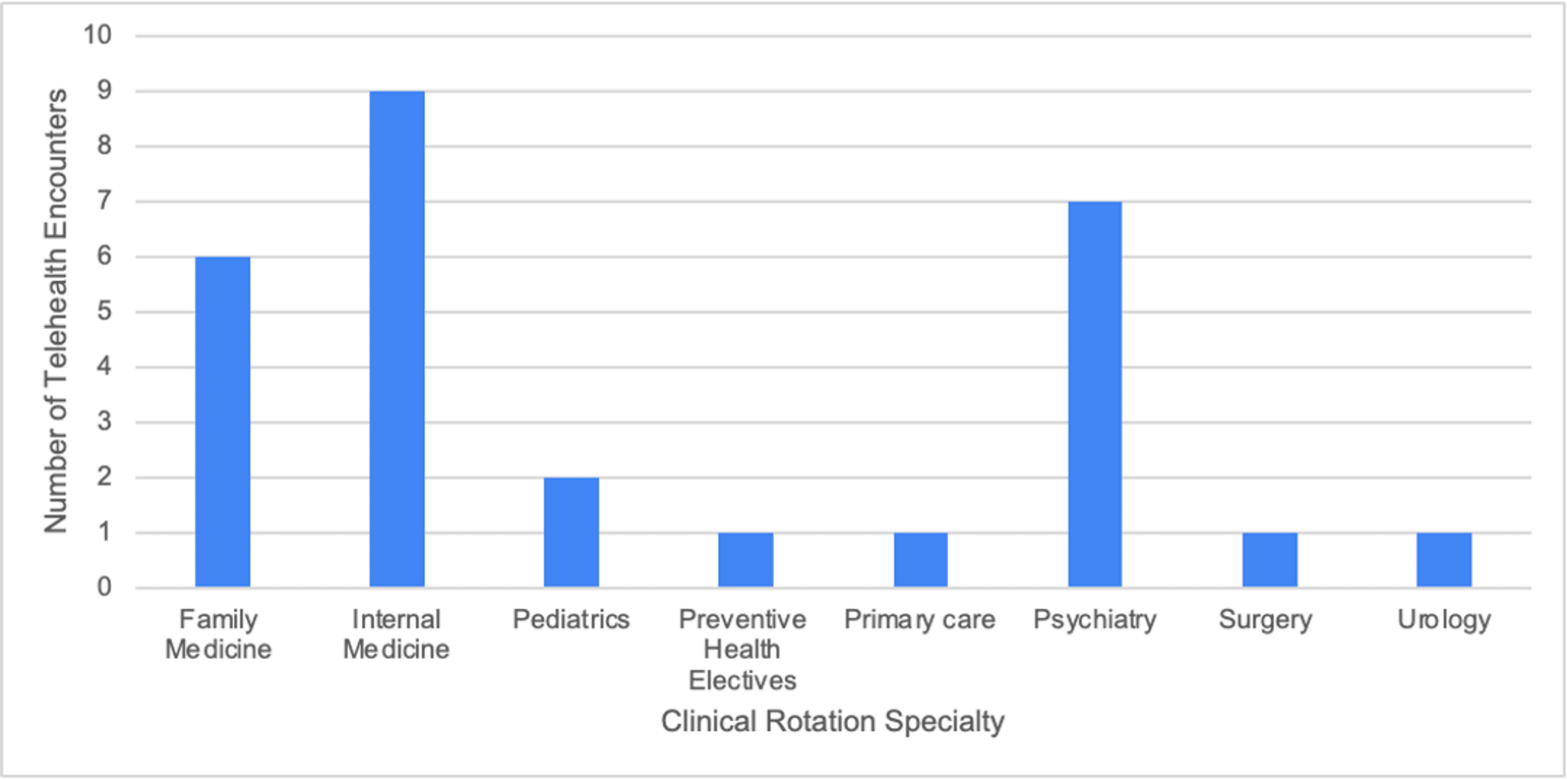
Number of telehealth encounters performed per clinical rotation specialty

As shown in Table 1, each survey question is listed in the proper order, along with the type of answer choices provided, which can be referenced while evaluating the rest of the figures. Specifically, Figure 1 illustrates the responses to question 3, where a majority of medical students reported not having been exposed to telehealth. However, their preceptors continued to conduct telehealth visits throughout their rotations, as shown in Figure 2, which is based on question 4. Figure 3 illustrates a list of specialties observed in ACOM's clinical rotations that performed the most telehealth visits, based on our medical student survey responses to questions 7 and 8.

**Table 1:** Telehealth survey questions

Question Number	Question	Question Type
1	What year of medical school are you in?	Multiple choice
2	Where is your clinical rotation site?	Fill in the blank
3	During the preclinical years of your medical school curriculum, did you receive any education on how to perform a telehealth visit?	Yes or No
4	During your clinical rotations, did your preceptor conduct any patient encounters through telehealth?	Yes or No
5	Did you receive any guidance/education on how to perform a telehealth visit from your rotation preceptors?	Yes or No
6	During your clinical rotations, did you conduct any patient encounters through telehealth?	Yes or No
7	If you stated yes previously, how many encounters?	Fill in the blank
8	If you stated yes previously, which rotation or specialty more commonly had telehealth visits?	Fill in the blank
9	Did you feel adequately prepared during those encounters based on your preclinical medical school curriculum?	Yes or No
10	Overall, do you think it would be beneficial to have more education on how to perform a telehealth visit during the preclinical years of your medical school curriculum?	Yes or No
11	Do you believe a physical exam can be adequately performed over telehealth?	Yes or No
12	During the patient encounter, I found the history-taking portion to be difficult to conduct over telehealth.	5-point Likert Scale
13	During the patient encounter, I found the physical exam portion to be difficult to conduct over telehealth.	5-point Likert Scale
14	During the patient encounter, I found the assessment and plan portion to be difficult to conduct over telehealth.	5-point Likert Scale
15	During the encounter, I found connecting with my patient to be difficult to conduct over telehealth.	5-point Likert Scale

**Figure 4:**
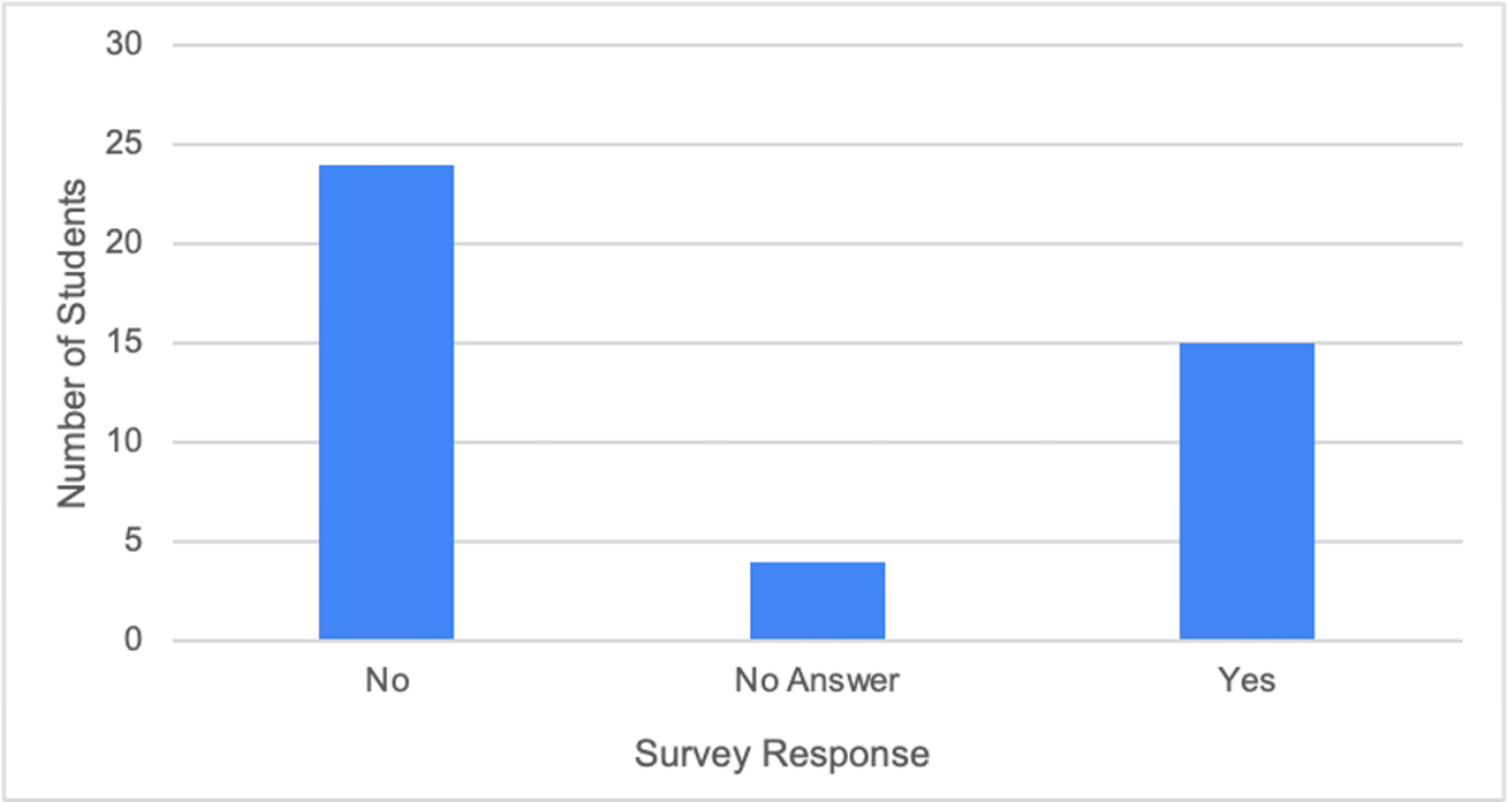
Whether the medical students felt prepared to perform a telehealth visit on their own during their clinical rotations

**Figure 5:**
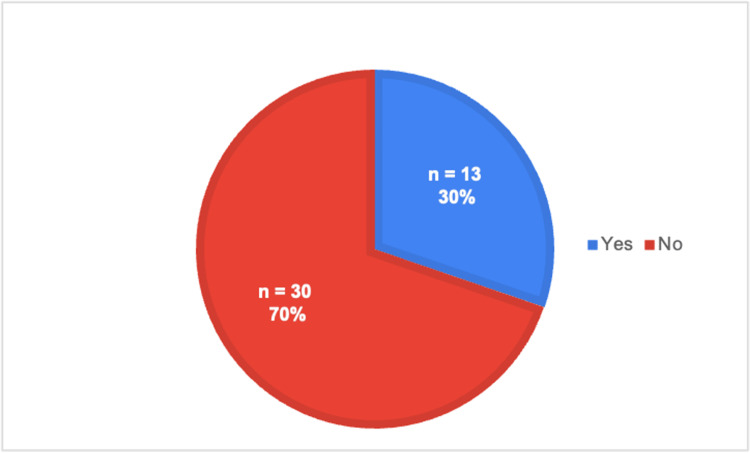
Whether the medical students received guidance/education from their preceptor on conducting telehealth visits

**Figure 6:**
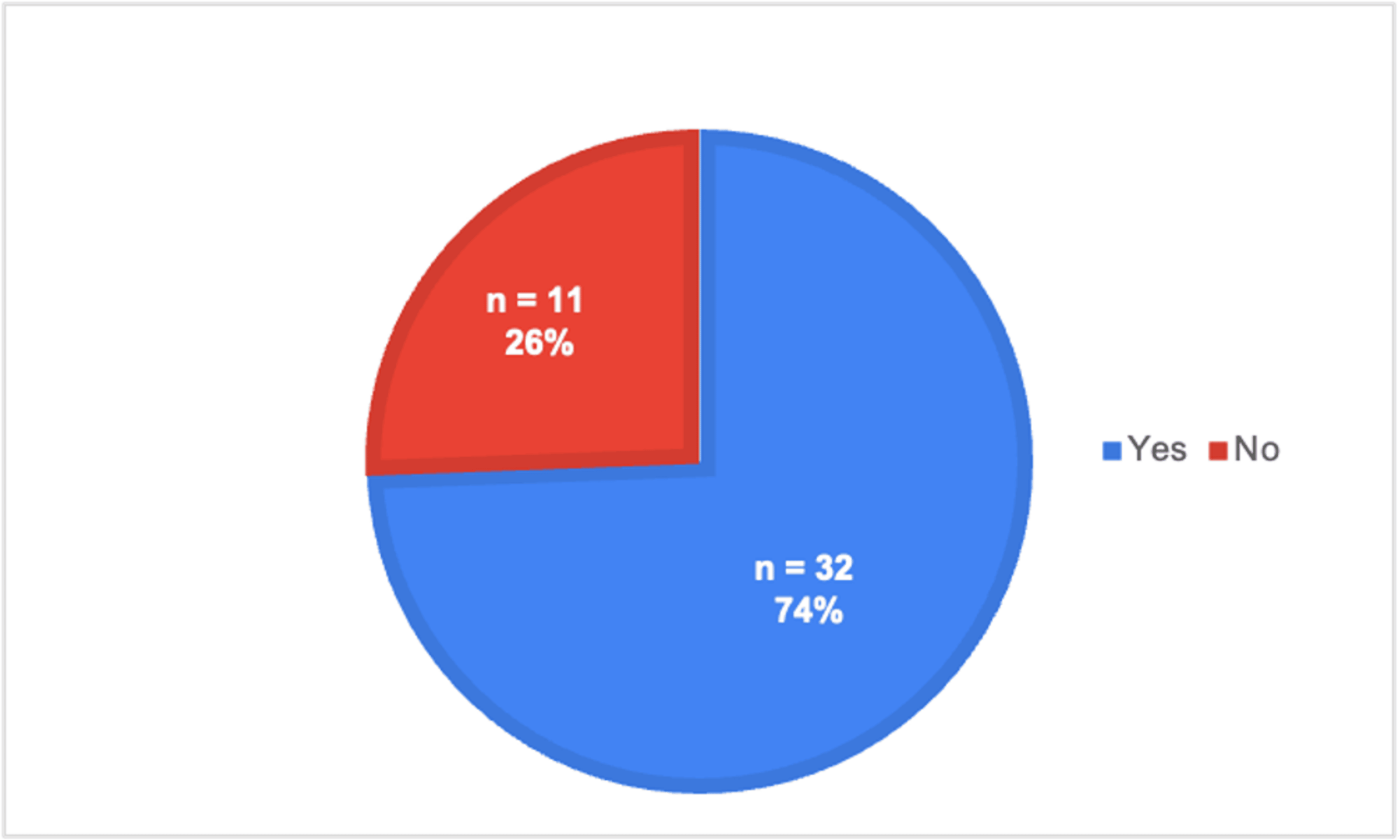
Need for telehealth education in the medical school curriculum

As seen in Figure 4, which is based on question 9, most students stated that they did not feel prepared to perform a telehealth visit independently during clinical rotations. In addition, most of those students did not receive guidance or education from their preceptors on how to conduct a telehealth visit properly or effectively, as seen in Figure 5 and the responses to question 5. In Figure 6, the responses are shown for question 10, indicating that a majority of medical students believed there was a need to incorporate telehealth education into the medical school curriculum.

**Figure 7:**
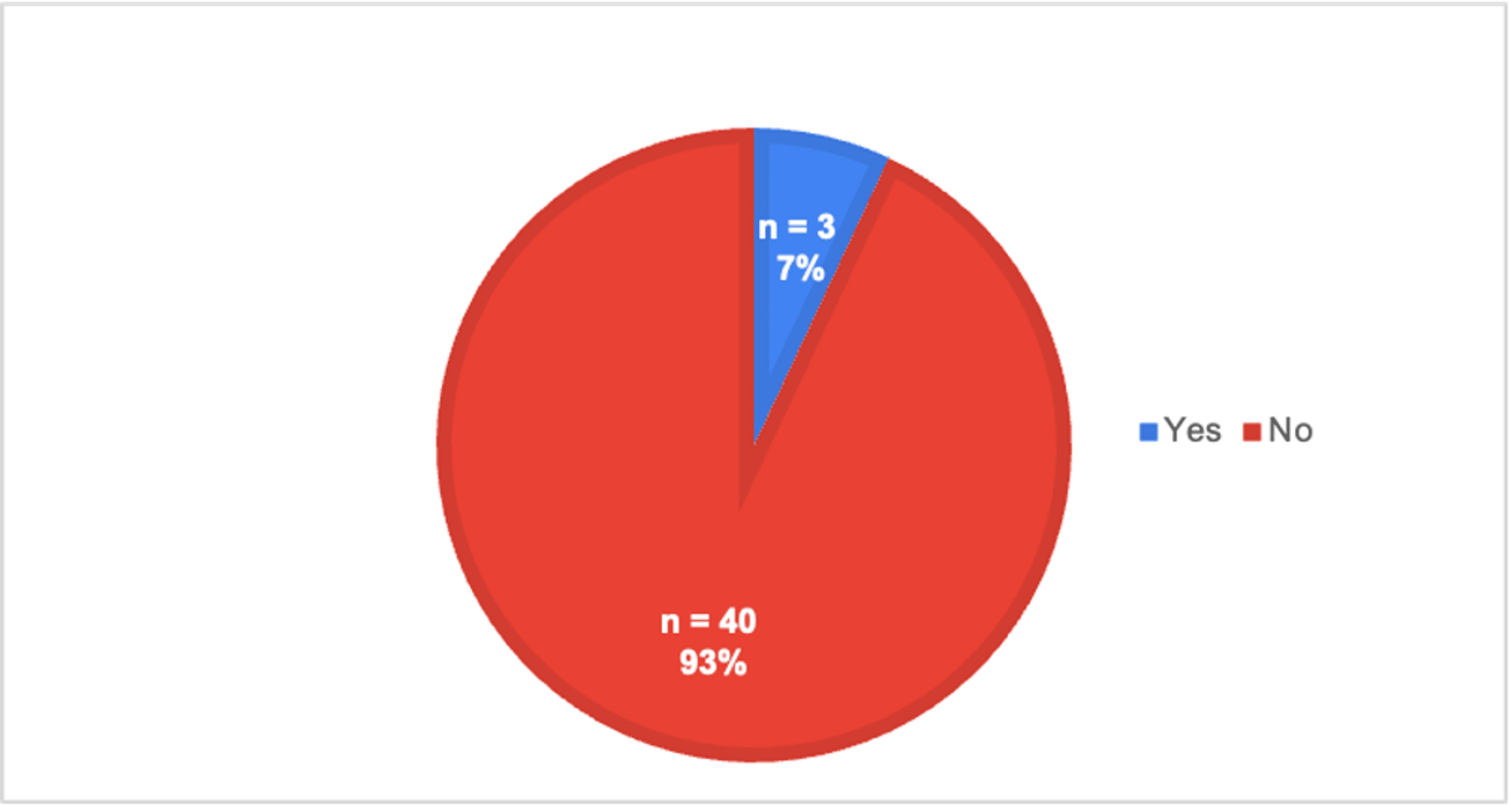
Whether a proper physical exam could be conducted over a telehealth visit

The last part of the survey aimed to determine which portion of the clinical encounter was the most difficult for the medical students who conducted a telehealth visit. As seen in Figure 7, almost all the responses indicated that the students did not believe they could conduct a thorough physical exam on their patient virtually, based on the responses to question 11.

**Figure 8:**
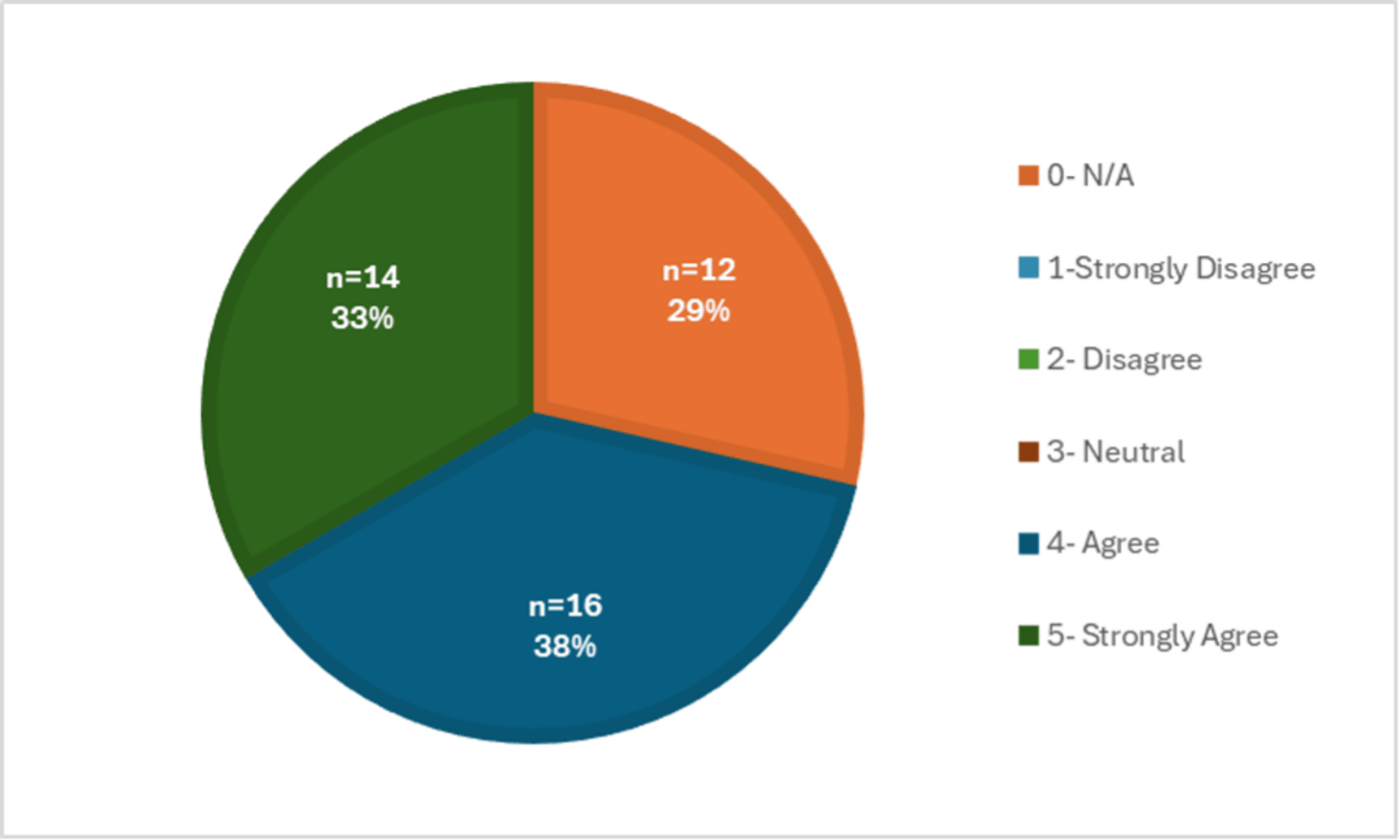
Difficulty of physical exam portion via telehealth

**Figure 9:**
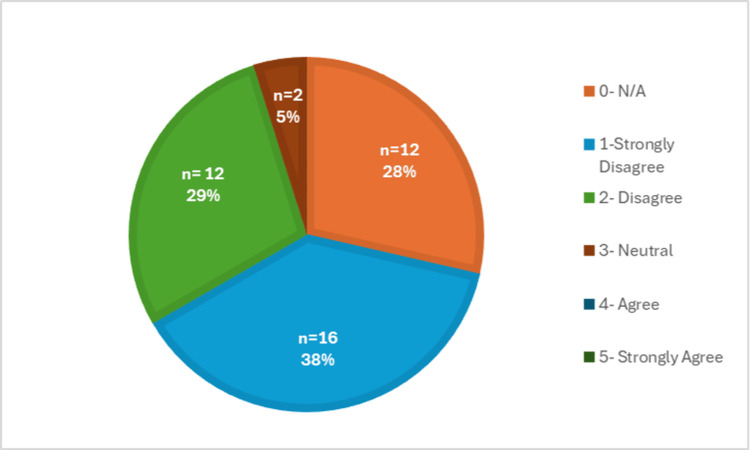
Difficulty of the history-taking portion via telehealth

**Figure 10:**
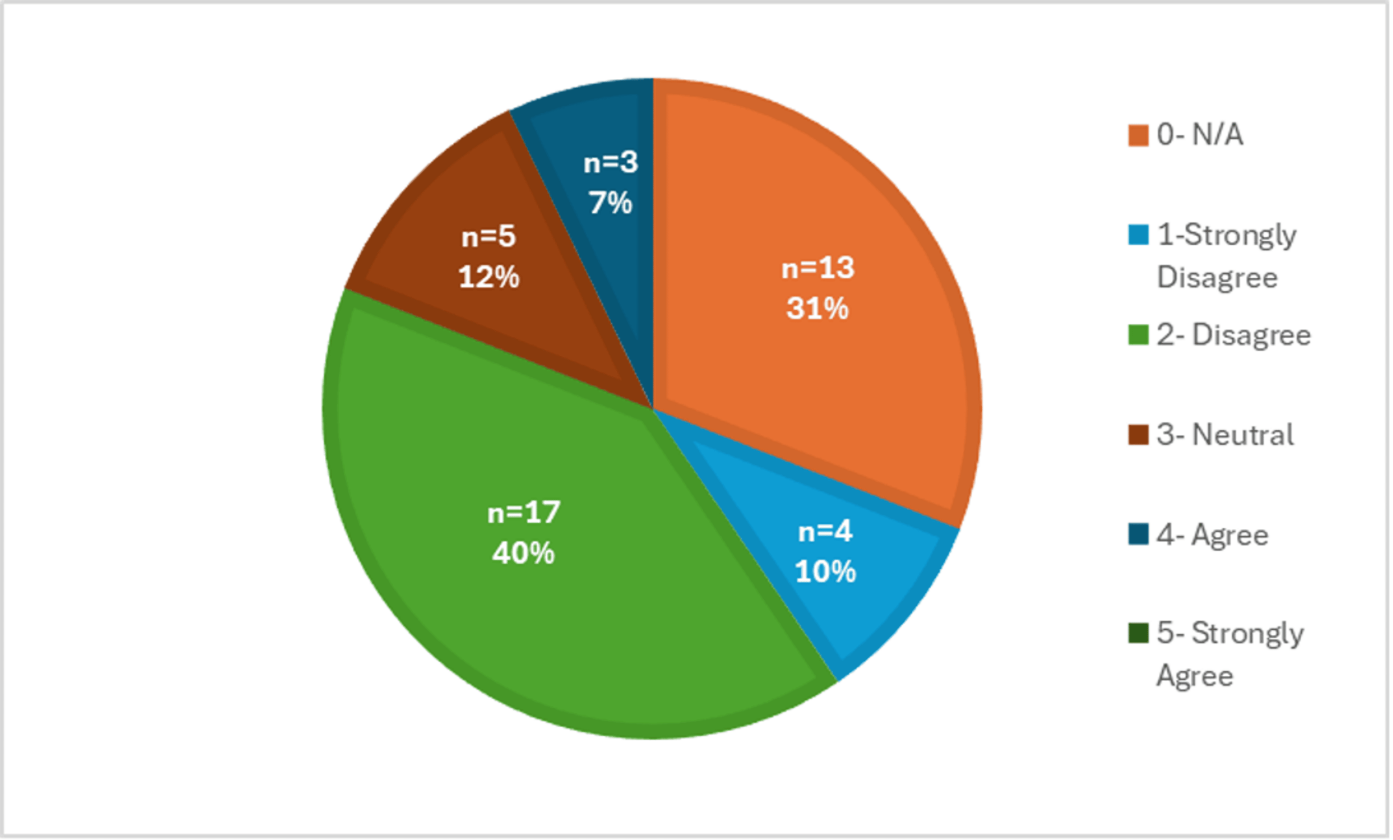
Difficulty of assessment and plan portion via telehealth

**Figure 11:**
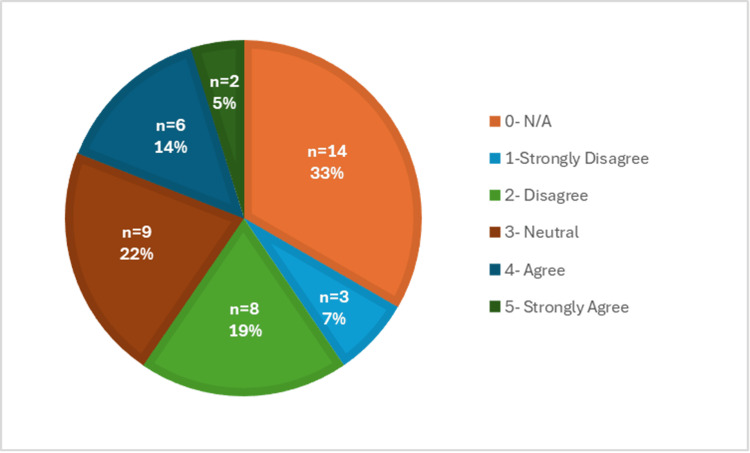
Difficulty of connecting with the patient via telehealth

The difficulty of completing a physical exam virtually was further supported by Figure 8, which showed that medical students completed a Likert scale indicating whether they found the physical exam to be the most difficult portion of the telehealth visit. Over 70% (n=30) of the responses agreed or strongly agreed with this statement. This part of the visit was rated the most difficult compared to the others, including history-taking (Figure 9), assessment/plan (Figure 10), and patient connection (Figure 11). Many of the responses found the assessment/plan to be not as difficult, with only 7% (n=3) of students agreeing with the statement (Figure 10). The least rated difficulty was seen in the history-taking portion of the telehealth encounter, with 67% (n=28) of students strongly disagreeing or disagreeing and 0% (n=0) agreeing or strongly agreeing with the statement (Figure 9). Each of these figures was based on the responses to survey questions 12 through 14.

Although patient connection was not rated as high in difficulty as the physical exam, there was still a mixed response based on the responses to question 15. As seen in Figure 11, 41% (n=17) of responses were either neutral, agreed, or strongly agreed with patient connection being difficult over telehealth.

## Discussion

Overall, the collected data revealed that the students who responded to the survey did not feel prepared to perform a telehealth visit; therefore, they recommended adding further telemedicine education to the preclinical curriculum. Additionally, the students also found the physical exam portion to be the most difficult to perform within the telehealth clinical encounter. In general, this was concerning because even though the students were thoroughly trained to conduct in-person visits, there are still many obstacles that arose when trying to convert this to a virtual atmosphere, which need to be addressed.

Specifically, Figure 2 shows that telehealth visits are still commonly conducted, even though we have passed the peak of the COVID-19 pandemic. Therefore, we can assume that telemedicine will continue to be prevalent in the healthcare field regardless of the medical specialty, as seen in Figure 3. Not surprisingly, family and internal medicine had some of the higher numbers. However, psychiatry also had one of the highest response rates. This is consistent with reports from students stating that the majority, if not all, psychiatry encounters were performed virtually. These results further demonstrate the continued adoption of telehealth methods, especially in primary care settings. However, even with telemedicine becoming more prevalent, many respondents stated that they did not feel prepared to conduct a telehealth visit on their own (Figure 4). Therefore, as shown in Figure 6, 74% of the students' responses indicated a need for telehealth education in the preclinical curriculum. This idea is not only supported by ACOM's medical students but also by many others who attend different schools located in various countries, who have had similar experiences and struggles with virtual medicine [7]. Specifically, a study at Northeast Ohio Medical University administered a survey to its medical students, which also found that 74% of the participants wanted telemedicine to be implemented in their medical school curriculum [8].

In addition, the survey also focused on identifying which aspects of the clinical encounter were the most challenging to perform virtually. In Figure 7, approximately 93% of the students reported that they felt they could not properly conduct a physical exam during a telehealth visit. This finding is also supported by other research articles, which have reported that medical students experience the same difficulty, especially with the physical exam section [9]. Therefore, we suggest that once a curriculum is created, there should be a higher emphasis on the physical exam portion of the encounter.

In many ways, we may have to change our definition of what constitutes a complete physical exam when conducting it via telehealth. As we know, there will always be limitations when in a virtual atmosphere, but it has been shown that with enough training, a telehealth physical exam can be equivalent to an in-person exam [10]. In general, certain portions of the clinical encounter, including history-taking and the assessment/plan, were found to have the least difficulty, which was demonstrated in Figures 9 and 10. Lastly, the responses regarding difficulty with patient connection during a telehealth visit have a mixed response, as shown in Figure 11. This was further supported by a recent study, in which medical students identified challenges, including conveying empathy and building rapport with their patients during telehealth visits [11]. Overall, this suggests that it would be beneficial to include education on how to develop patient connections via telehealth to strengthen students' abilities and confidence in this area. Patient connection is vital as it allows physicians to form a trusting relationship with their patients, which leads to effective communication and better health outcomes.

Considering the current and expected demand of telehealth, dedicated telehealth training is needed in the preclinical and clinical years for students to feel confident and competent in delivering quality healthcare via virtual communication. In the pre-clinical years, instruction could be delivered in various methods. In the following proposed method, telehealth education would be implemented into the course that teaches primary clinical skills. A three-week block in the second year will be dedicated to telehealth instruction and will be laid out as follows. During the first week of the block, in-house-created online modules and a lecture-based series will be used to teach the students about telehealth principles and how to properly incorporate them in clinical practice [12]. During the second week of the telehealth block, simulation workshops or labs will be held, providing students with the opportunity to practice telehealth visits with standardized patients. During these simulation practice sessions, physician faculty will be present to guide and assist. It was found that performing these simulations for telehealth visits was highly feasible and had high participant satisfaction, further supporting their implementation [13]. Lastly, in the third week, students will be given a telehealth written examination and an objective structured clinical exam (OSCE) to demonstrate mastery of the material. Alternatively, a telehealth lesson could be incorporated for each system block taught in primary clinical skills. This way, students can learn and practice performing various physical exams on the body systems virtually. This variation will also include simulation workshops, examinations, and OSCE evaluations. It was found that simply adding an online-based module for medical students has shown an increase in their confidence in conducting telehealth visits [14].

Furthermore, a study at Johns Hopkins University School of Medicine showed that students who received the telehealth medicine curriculum had a notably higher mean score on their OSCE telemedicine case than those who did not receive the curriculum [15]. Therefore, if the entire three-week block cannot be implemented at once, then adjusting the curriculum by incorporating an online-based telehealth module could greatly improve the efficiency and quality of care for future patients who decide to meet their physician virtually.

Additionally, it is also crucial that the faculty themselves are properly trained to teach and conduct these lectures and evaluations. In the referenced article, multiple methods were proposed to aid faculty in their continuous education on telemedicine, including interactive environments and online-based modules [16]. A proposed method to educate the faculty could be applied in the following manner. Faculty would be educated through an interactive lecture series that aligns with the national telehealth standards (Association of American Medical Colleges (AAMC) and Accreditation Council for Graduate Medical Education (ACGME)) [16]. Following the lecture series, the American Medical Association (AMA) modules and reading material will be completed by the faculty. The AMA telehealth module, "Telehealth and Team-Based Care," and the AMA's Telehealth Implementation Playbook will be required to be completed as part of faculty training. Furthermore, faculty will be educated on how to facilitate an OSCE that best examines the students' competency in telehealth. During clinical years, it is expected that preceptors will introduce and incorporate virtual care into their instruction. Preceptors should be expected to monitor the student's effectiveness via telehealth until the preceptor feels comfortable with the student acting with only indirect supervision to increase efficiency [17].

Our survey was distributed to only one medical school (ACOM), and of that, only 11.6% of the third- and fourth-year students participated. Therefore, we cannot fully assess the extent of the need to include telehealth in the curriculum. However, the 43 responses we received can serve as a preliminary indicator of the perceived need within the class. Possible reasons for the low response rate could be that the survey was sent to students nearing the end of an academic year, and they were too "busy" to answer the survey or even see the email with the survey in their inbox. Additionally, because the survey was self-developed and had a limited response rate, there is a possibility of response bias where students with stronger opinions or a particular interest in telehealth might have been more likely to participate, which could have influenced the results. It is also important to note that for the four Likert scale questions assessing the difficulty of various aspects of the telehealth encounter, about one-third of the responses were marked as "N/A." The results for these questions might have varied depending on each student's level of access to patients at their respective clinical rotation sites. Another potential limitation is that the data were collected from a single institution. The experiences, curriculum structure, and clinical opportunities at ACOM may not reflect those at other medical schools, particularly those in different geographic regions or with differing resources and telehealth infrastructures. This could limit the generalizability of our findings to a broader medical student population. Including participants from multiple institutions in future studies might help provide a more diverse and representative understanding of medical students' perspectives. Furthermore, while this survey provides useful preliminary insights, future research might benefit from incorporating more in-depth qualitative analysis to better understand students' experiences and specific challenges with telehealth. Although we can reasonably speculate that enhanced training will lead to greater competency in telehealth practice, this assumption also needs to be formally assessed and validated in future research to confirm its effectiveness.

## Conclusions

Our findings indicate that most surveyed students felt unprepared to conduct telehealth visits and strongly supported integrating telemedicine education into the curriculum. In addition, the medical students who responded found the physical exam to be the most difficult part of the clinical encounter to conduct virtually, which should be emphasized in curricular changes. While it is highly recommended that telehealth education be incorporated into the medical school curriculum, due to the limitations of our study, we caution against generalizing our findings to other schools. The key recommendations include integrating online modules for foundational knowledge, simulation-based training for virtual clinical skills, and OSCEs for competency assessment. Additionally, faculty training must be prioritized to ensure the effective delivery of telehealth instruction. Implementing these changes will enhance medical students' confidence and competency in telehealth, ultimately improving patient access to quality virtual care. Future studies should evaluate the effectiveness of these interventions in preparing students for digital healthcare delivery.

## Figures and Tables

**Figure 12:**
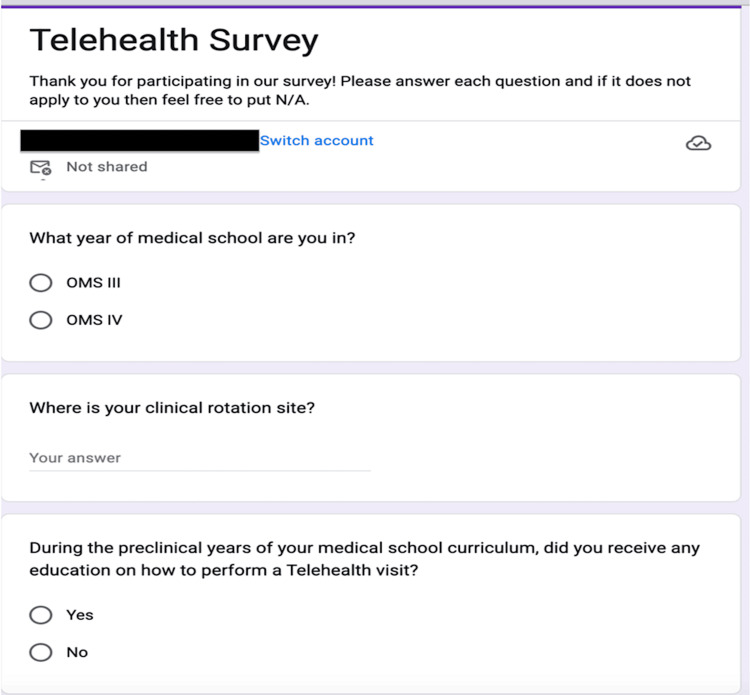
Telehealth survey as presented on Google Forms including Q1-3

**Figure 13:**
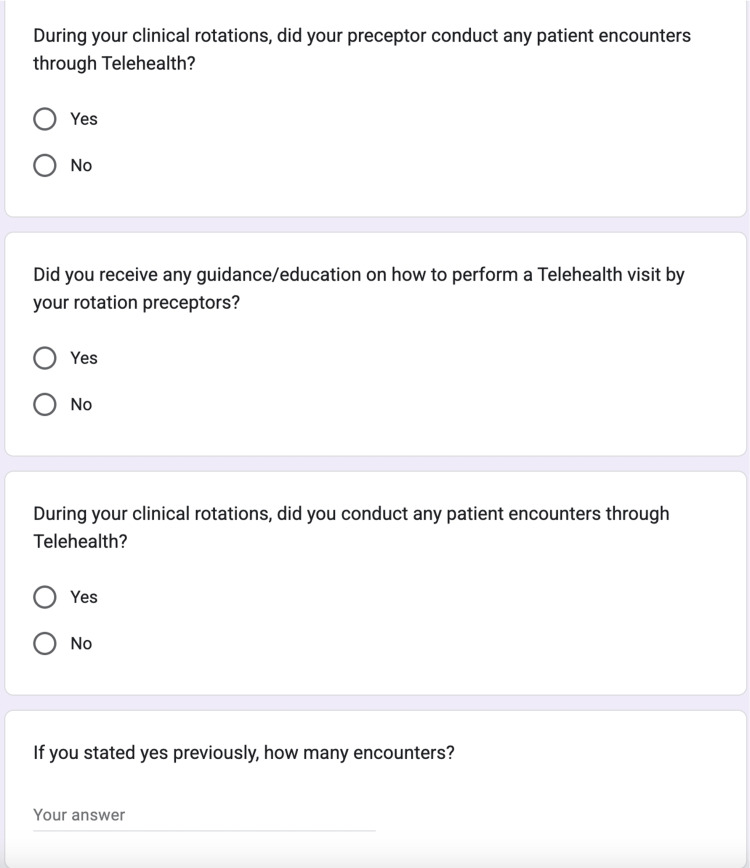
Telehealth survey as presented on Google Forms including Q4-7

**Figure 14:**
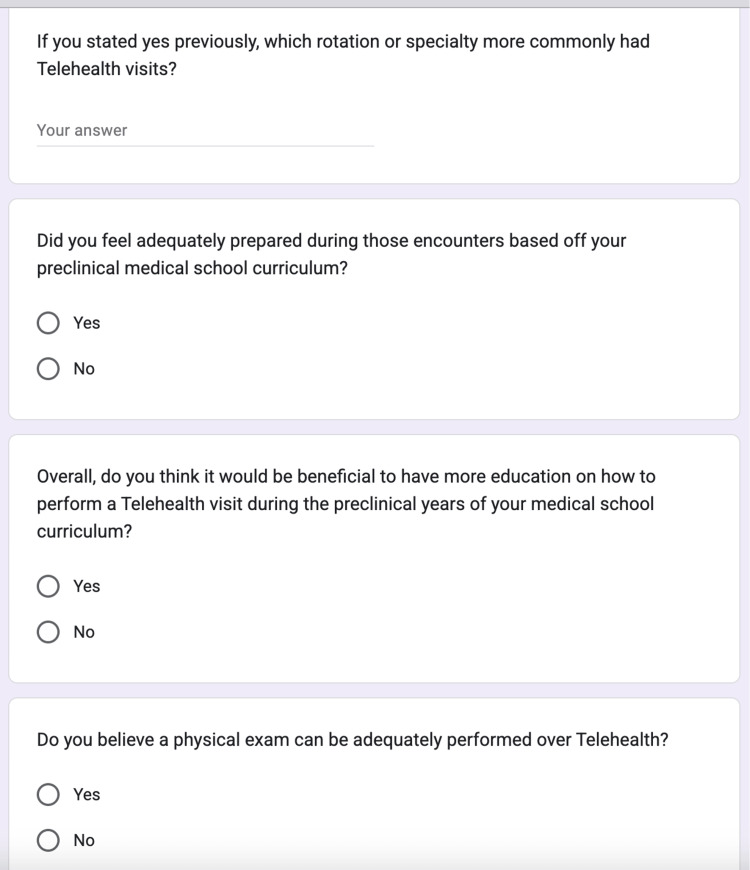
Telehealth survey as presented on Google Forms including Q8-11

**Figure 15:**
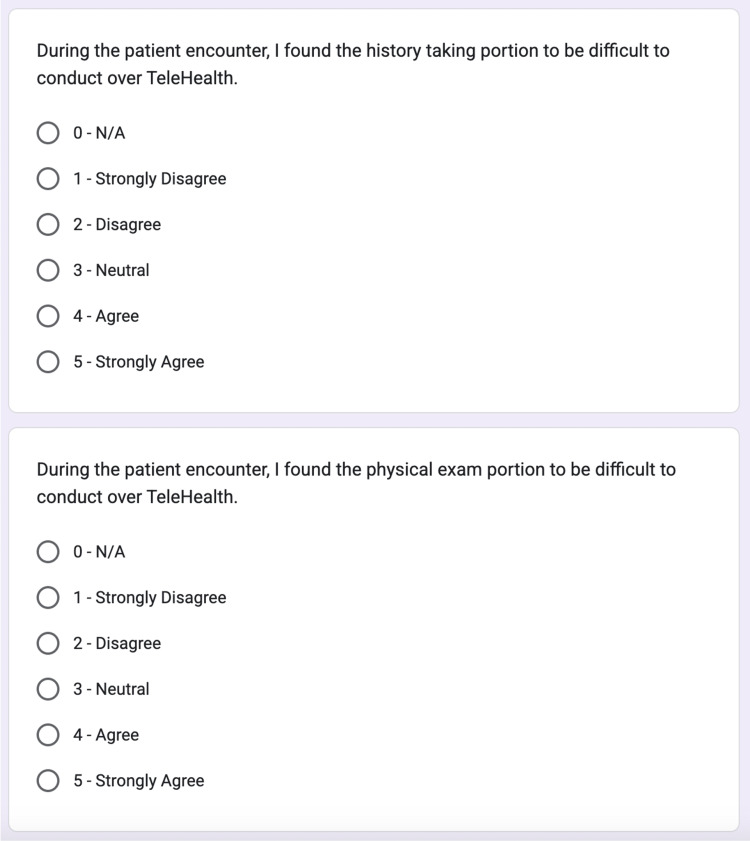
Telehealth survey as presented on Google Forms including Q12-13

**Figure 16:**
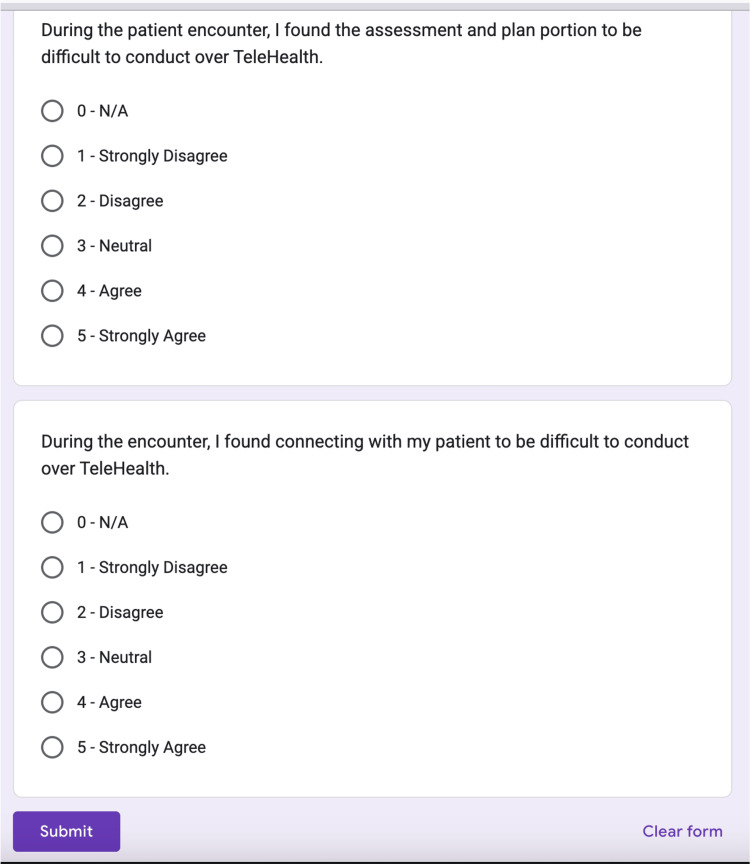
Telehealth survey as presented on Google Forms including Q14-15

## Appendices

Figures 12-16 depict the telehealth survey that was created and distributed as mentioned in earlier sections of this research article.

Survey Link:

https://docs.google.com/forms/d/e/1FAIpQLSdKEJrVFItC8hLbri_jEdMZZKQgmbTLB_sCmmba-nUkkjOnnA/viewform?usp=sf_link
